# Effects of the Plastic of the Realistic GeePS-L2S-Breast Phantom

**DOI:** 10.3390/diagnostics8030061

**Published:** 2018-09-01

**Authors:** Tomas Rydholm, Andreas Fhager, Mikael Persson, Shireen D. Geimer, Paul M. Meaney

**Affiliations:** 1 Department of Electrical Engineering, Chalmers University of Technology, 41258 Gothenburg, Sweden; andreas.fhager@chalmers.se (A.F.); mikael.persson@chalmers.se (M.P.); paul.m.meaney@dartmouth.edu (P.M.M.); 2 Thayer’s School of Engineering, Dartmouth College, Hanover, NH 03755, USA; shireen.geimer@dartmouth.edu

**Keywords:** breast cancer, microwave imaging, phantom, tomography

## Abstract

A breast phantom developed at the Supelec Institute was interrogated to study its suitability for microwave tomography measurements. A microwave measurement system based on 16 monopole antennas and a vector network analyzer was used to study how the S-parameters are influenced by insertion of the phantom. The phantom is a 3D-printed structure consisting of plastic shells that can be filled with tissue mimicking liquids. The phantom was filled with different liquids and tested with the measurement system to determine whether the plastic has any effects on the recovered images or not. Measurements of the phantom when it is filled with the same liquid as the surrounding coupling medium are of particular interest. In this case, the phantom plastic has a substantial effects on the measurements which ultimately detracts from the desired images.

## 1. Introduction

Microwave tomography is a method of imaging with potential for applications over a vast range of fields. Medical applications are emerging in areas such as bone-density measurements [1], brain imaging [2,3], cardiac imaging [4] and breast-cancer diagnosis [5,6] to name a few. Previous studies have often been limited to idealistic simulations but the high dynamic range of modern vector network analyzers (VNAs) makes it possible to study real data over a broad frequency band.

Several systems for microwave imaging of breast cancer have now reached clinical tests. Techniques are broadly divided into three categories: (a) radar; (b) holographic; and (c) tomographic techniques. Radar based systems have been developed at the University of Bristol [7], University of Calgary [8], and McGill University [9] and have matured to the phase of phantom experiments and clinical trials. Holographic approaches have been introduced more recently and have shown promise in limited phantom studies [10]. 2D and 3D tomographic, or inverse-scattering methods, have been studied extensively in simulation studies [11,12] with only a limited number advancing to the stage of phantom experiments or clinical studies [13,14,15]. This study focused on phantom experiments utilizing a tomographic system built at Chalmers University of Technology, based on the concepts of the system developed at Dartmouth College [6].

Among women, breast cancer is the single most common type of cancer [16]. It has been estimated that over 260,000 new cases will occur in the US during 2018 and that 41,000 women will die from the disease [16]. Early detection and treatment is crucial for a likely recovery. The development of new technology for diagnosis, such as microwave tomography, could potentially contribute to a significant reduction of these numbers. X-ray based mammographic screening is the current gold standard. This technology has the advantage of high resolution; however, in dense breasts, it can be particularly difficult to distinguish between malignancies and benign lesions and normal tissue [17]. Due to the differences in dielectric properties between different tissue types, microwave imaging could be beneficial [18,19,20]. These differences originate primarily from differences in water content between tumors and regular adipose and fibroglandular tissues [21]. More recently, studies indicate that bound-water features may also contribute to these differences [6].

During the development and evaluation of microwave tomographic systems, measurements on realistic phantoms are vital. Phantoms are models of body parts or organs that have been designed to mimic the properties of their biological counterparts, not just in shape and size but also in physical properties. For the case of microwave tomography, the permittivity and electrical conductivity dictate the field propagation behavior as governed by Maxwell’s equations. Different materials have been considered as suitable substitutes for biological tissue. Examples of such substitutes are gels [22], Triton X-100 [23], rubber-carbon mixtures [24] and glycerin [25]. Common methods of modifying the properties include mixing liquids with different ratios of water and varying the salt content. However, there is considerable debate over what ranges of values of dielectric properties are most representative of breast tissue. For example, Sugitani et al. [18] reported values for the relative permittivity measured at 1.5GHz of 45, 25 and 7 for malignant, fibroglandular, and adipose tissue, respectively. Lazebnik et al. [26] studied the dielectric properties of normal and malignant breast tissue for different ratios of fibroglandular to adipose tissue at a wide range of frequencies. Other recent studies include those by Martellosio et al. [19], Cheng and Fu [20], and Gabriel [27]. Utilizing mixtures of glycerin and water produces substantial variations for designing liquids of different properties [25].

Phantoms play an even more important role when it comes to breast cancer since there are no suitable animal models compared with other anatomical sites. Phantom experiments are a good way to test and validate a system before clinical evaluation after transitioning from just simulation studies. Simulations are a necessary and important tool in the early process of developing a system, but, to reach clinical studies and ultimately a functional system, controlled experimental measurements using actual data are essential.

Phantoms used for the development of breast cancer diagnosis should replicate the complex geometry of a human breast. A human breast is mainly comprised of two different tissue types: adipose and fibroglandular. Due to different ratios of water, fat and protein, these tissues show different dielectric properties and hence it is possible to distinguish them from each other [21].

A phantom with simplistic geometry can easily be fabricated by using canonically shaped inclusions representing the fibroglandular tissue inside a larger vessel of liquid with properties mimicking adipose tissue. However, to represent the complex geometry of an actual human breast, more sophisticated phantoms are being developed. Examples of more realistic phantoms include the ones developed by Burfeindt et al. at the University of Wisconsin [28], Joachimowicz et al. at the Supelec institute [29] and similar ones designed by Herrera et al. at the University of Manitoba [30].

In this investigation, we expand on our previous study of the GeePS-L2S phantom [31] developed at the Supelec institute. In that report, we were able to recover good images of the phantom using a tomographic system. However, there is good reason to believe that the high plastic content of the 3D-printed phantom boundaries prevents the interior of the phantom from being more accurately recovered due to the high contrast scattering from the relatively thick, low dielectric plastic interfaces. In this paper, we investigate more thoroughly how the plastic impacts the imaging.

The two separate chambers of the phantom (corresponding to adipose and fibroglandular tissue, respectively) are studied and imaged one at a time. For each chamber, measurements are performed with both the ordinary tissue-mimicking liquid and the same liquid as the surrounding coupling bath. The reconstructed images are then compared to study the effects of the plastic. An MRI scan of the phantom was performed to fully quantify the plastic shape and size at different layers.

## 2. Materials and Methods

We have previously demonstrated that our microwave-tomography system is capable of imaging the GeePS-L2S phantom [31]. However, the recovery of the phantom interior was less optimal. In the previous study, a simpler cylindrical phantom of comparable size and comprised of the same tissue mimicking liquids was also imaged for comparison. In that case, the fibroglandular-tissue mimicking inclusion was clearly distinguishable. One hypothesis to explain this is that the high plastic content of the GeePS-L2S phantom was significantly contributing to the overall dielectric property distribution and subsequently adversely influencing the images.

This study focuses on the plastic shells of the GeePS-L2S phantom. The two shells of the phantom are interrogated separately and reconstructions are performed for the shells filled both with their ordinary tissue mimicking content and with the surrounding coupling liquid. In addition, the actual measurements are also investigated to confirm that the plastic effects are evident in the raw data and not just due to inadvertent features of the reconstruction algorithm.

### 2.1. The System

**Figure 1:**
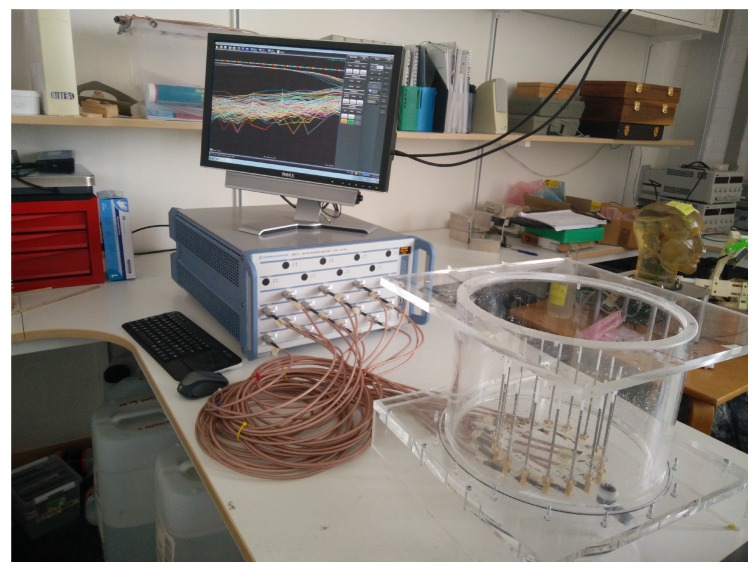
The measurement system used for the study. To the left is the VNA and to the right is the immersion tank containing the antennas and the coupling liquid.

The measurement system is described in [32] and a photograph is shown in Figure 1. It is based on sixteen monopole antennas arranged in a circle with a diameter of 15.2cm surrounding the target region. The antennas are connected via coaxial cables to a sixteen-port VNA (Rhode & Schwarz ZNBT8) so that no external switching matrix is needed. The VNA operates over a frequency range from 9 to 8.5GHz and has a dynamic range of more than 130dB over the full operating frequency range. The channel-to-channel isolation is greater than 150dB.

A cylindrical tank surrounds the antennas, which is filled with a mixture of 80% glycerin and 20% water (volume percentage). This coupling medium has two purposes. Since a high permittivity contrast contributes to large scattering, the liquid concentration is chosen to lower the contrast between the breast and its surrounding environment. The second involves its attenuating properties which are exploited to suppress effects from multi-path signals and surface waves [6,33]. For calibration, a set of measurements in the homogeneous coupling liquid is performed as a reference which is then subtracted from the measurements of the actual phantom submerged in the liquid. In this manner, the measured difference or projection is effectively only due to the target being present in the immersion tank.

Measurements are performed at multiple frequencies between 1 and 1.9GHz using an IF bandwidth of 10Hz and an output power of 0dBm. Averaging was performed over 10 measurements. The complex-valued *S*-parameters are then collected and utilized in the reconstruction algorithm described in [34].

### 2.2. The Phantom

**Figure 2:**
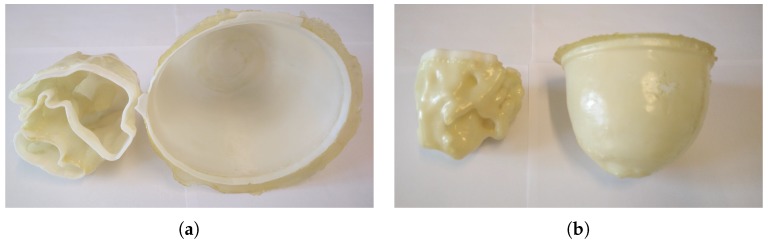
The two shells of the GeePS-L2S phantom: (**a**) a top-down view; and (**b**) a view from the side. To the left is the inner fibroglandular shell. To the right is the outer adipose shell.

The GeePS-L2S-breast phantom is a 3D printed plastic phantom made out of Acrylonitrile butadiene styrene (ABS) derived from an MRI-based numerical phantom available from the UWCEM Numerical Breast Phantom Repository [35] and is shown in Figure 2. It consists of two parts, each forming a chamber corresponding to the different tissues of a real breast, i.e., the fibroglandular tissue for the inner zone and the adipose tissue for the outer zone. Different research groups around the world are currently testing the phantom in their respective imaging systems [31,36,37,38].

**Figure 3:**
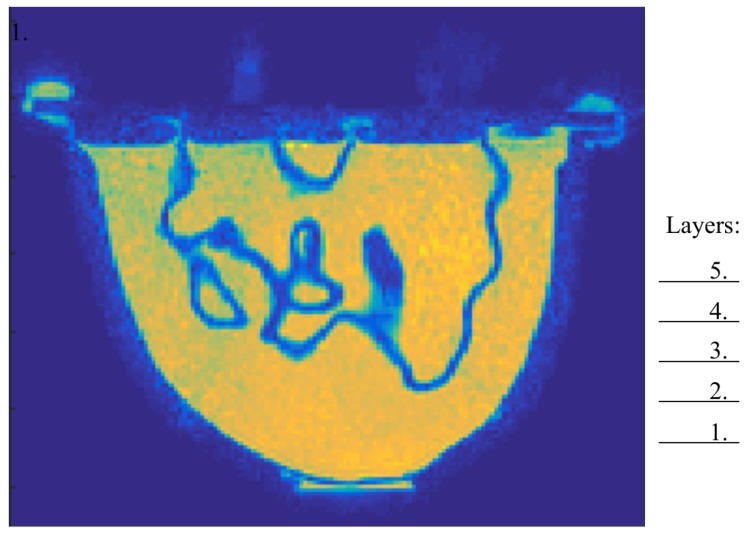
Cross section of an MRI scan of the GeePS-L2S phantom. The positions of the five imaging planes are marked out.

To present an accurate visual rendering of the phantom interior and to calculate the amount of plastic of each imaged layer, an MRI scan was performed (water was used as the contrast liquid). Figure 3 shows the MRI image for a single transversal plane through the phantom. It can clearly be seen that the low permittivity plastic forms a significant proportion of the overall phantom.

The plastic forms a 1.5mm thick interface between the different regions [38]. The wrinkled surface of the interior chamber implies that the effective thickness of the wall is probably considerably larger in many planes. Wide frequency-range data were not provided for the ABS-plastic but it has been reported to have a relative permittivity of roughly 3 at 2.4GHz [38], which is significantly lower than that for the liquids used in this experiment. The combination of its thickness and high contrast with the relevant liquids could act to skew the desired measurements.

A mixture of 88% glycerin and 12% water was used for the adipose region, and a corresponding ratio of 72:28 was used for the fibroglandular region. While there is considerable debate within the community as to optimal breast-tissue properties and, subsequently, what the most suitable phantom material recipes are, the glycerin:water mixtures allow for easy variability and a freedom to choose from a wide range of dielectric properties [25]. Given that this study focuses on the differences between when the plastic is present or not, the glycerin–water mixtures are suitable liquids for this experiment. The two shells were studied individually, filled with their corresponding tissue mimicking liquid. To investigate the effects of the phantom plastic, image reconstructions were also performed for when these liquids were exchanged for the surrounding coupling bath. This provides the opportunity to assess the effects of just the shells. It is also worth noting that pure adipose fat in certain studies has been reported to have a lower permittivity than that for the 88:12 mixture, corresponding to a higher glycerin ratio [27]. In this study, adipose tissue corresponding to a radiographically scattered breast has been considered with properties based on clinical studies [39]. The choice of liquid can here be altered to some extent to account for different radiographical densities.

Due to the 3D variability nature of the phantom, it is also informative to explore whether different layers of the phantom are reconstructed equally well. The exposed parts of the monopole antennas are 3cm long. The effective imaging plane corresponds to the center of this but in practice provides a weighted average of contributions from parts slightly below and above this plane. The first layer, corresponding to the nipple being placed in the imaging plane, was performed followed by layers spaced 1cm apart from each other. Five layers were imaged until the fixture, from which the phantom was suspended, contacted the antennas.

### 2.3. Inverse Problem

The measurements consist of 240 complex data (16 transmitters by 15 receivers per transmitter) which describe the shifts in amplitude and phase compared to the reference. The data for the reflections ($S_{i,i}$) were not used. These data were fed into the Gauss–Newton iterative reconstruction algorithm. The algorithm converges towards an appropriate image based on minimizing the differences between the measured amplitudes and phases compared with that for the forward solutions computed at each iteration. Incorporation of the log transform and a reduced step size at each iteration have been instrumental in eliminating the need for a priori information [34].

The algorithm can be divided into two steps. First, 50 iterations of a smoothed Levenberg– Marquardt regularization are performed. This is followed by 20 iterations of a Tikhonov regularization with a Euclidean distance penalty term where the final image of the first step is used as the initial estimate of the latter. The algorithm is further described in [34].

During the Levenberg–Marquardt step, the cost function is written as:

(1) $$f_{LM}\left(k\right)=\left|\right|\Gamma^{m}-\Gamma^{c}\left(k^{2}\right){\left|\right|}^{2}+\left|\right|\Phi^{m}-\Phi^{c}\left(k^{2}\right){\left|\right|}^{2}$$

Here, Γ and Φ are logarithmic magnitudes and phases, and the superscripts *m* and *c* denote the measured and computed values, respectively. *k* is the wave number which can be expressed in terms of the relative permittivity $\varepsilon_{r}$ and conductivity σ through

(2) $$k^{2}=\omega \mu_{0}\varepsilon_{0}\varepsilon_{r}+j\omega \mu_{0}\sigma .$$

Here, ω is the angular frequency and $\varepsilon_{0}$ and $\mu_{0}$ are the free-space permittivity and permeability, respectively. The weighting between the two terms of Equation (1) have, in accordance with a previous study [40], been set to unity.

The cost function for the Tikhonov step is similar but carries an extra penalty term:

(3) $$\begin{array}{cc} f_{T}\left(k\right)= & \left|\right|\Gamma^{m}-\Gamma^{c}\left(k^{2}\right){\left|\right|}^{2}+\left|\right|\Phi^{m}-\Phi^{c}\left(k^{2}\right){\left|\right|}^{2}\\ & +\lambda ||k^{2}-k_{init}^{2}{\left|\right|}^{2} \end{array}$$

The notation is the same as in Equation (1) with the addition of λ being an empirically determined regularization parameter and $k_{init}^{2}$ being the intermediate solution that was obtained from the Levenberg–Marquardt step.

### 2.4. Measurements

**Figure 4:**
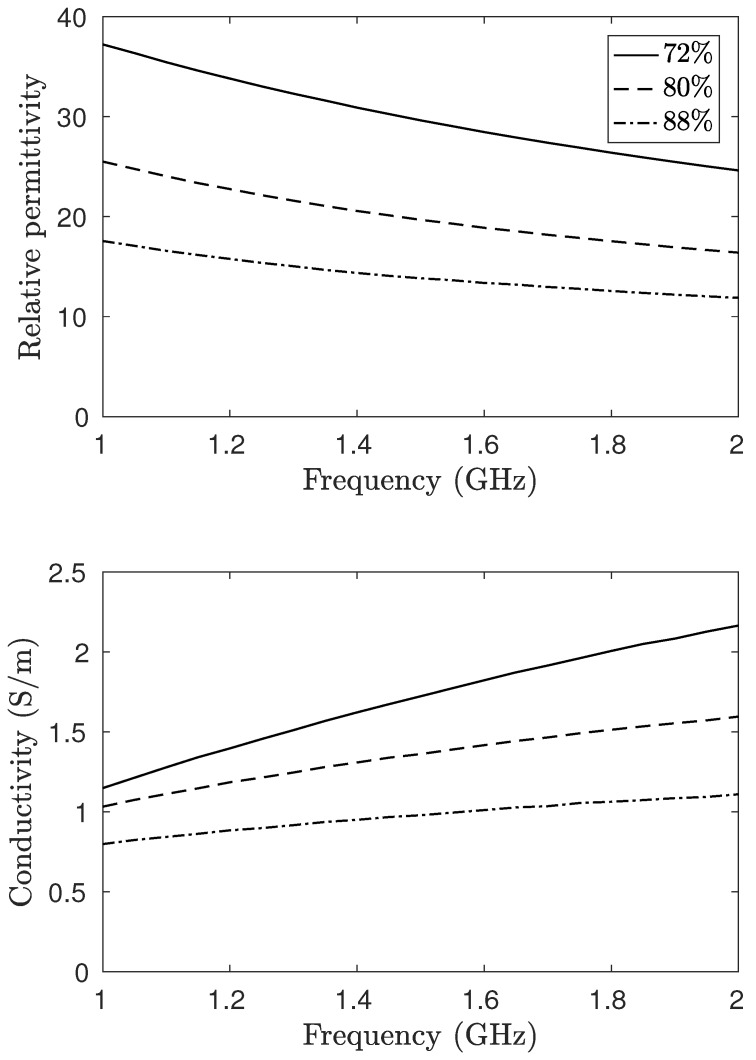
Dielectric properties of the associated glycerin–water mixtures as function of the frequency. This should be compared to the plastic having a permittivity of roughly 3 at 2.4GHz.

Images were reconstructed at five frequencies in the range from 1100 to 1900MHz. For frequencies lower than 1GHz, the inherent liquid attenuation was too low to fully suppress unwanted effects of surface waves and multi-path signals [6]. To minimize the occurrence of image artifacts due to surface reflections, the surface level of the coupling liquid was kept constant at 3cm above the antenna tips at all measurements. Three different mixtures of glycerin and water were used for the phantom. For the adipose tissue, the glycerin to water ratio was 88:12; for the fibroglandular, it was 72:28. In addition, to study the effects of the plastic, a mixture of the same ratio as the coupling liquid (80:20) was used. The dielectric properties of these mixtures over the operating frequency range can be found in Figure 4. These ratios have been determined to be good representations for a scattered breast (88:12) and fibroglandular tissue (72:28) in a previous study [39].

In total, four measurements series are performed. Two series are performed where just the outer shell is used (the adipose region) and two series when only the inner shell is used (the fibroglandular region). The contents of the phantom for these series are presented in Table 1.

For example, by comparing the data for series C and D, it is possible to determine the effects of the plastic of the fibroglandular part alone. For each of these series, five layers of the phantom were reconstructed, starting from the nipple and moving in increments of 1cm towards the chest wall.

## 3. Results

In the first section, we examine the amplitude and phase projections of the measured signals. In this case, the projections refer to the calibrated case where the measurements (in dB for amplitude and degrees for phase) for the homogeneous bath case are subtracted from those for the different phantom cases. Since the reconstructions of associated images are directly related to the actual measurements by virtue of the algorithm’s minimization process, trends observed in the measurements will also be visible in the images. For this analysis, the former is especially relevant since it is effectively presented without associated features of the reconstruction algorithm. The recovered images are shown in Section 3.2 along with concomitant MR images of the different imaging planes for comparison with observations of the measurement data and actual geometrical features.

### 3.1. Amplitude and Phase Projections

**Figure 5:**
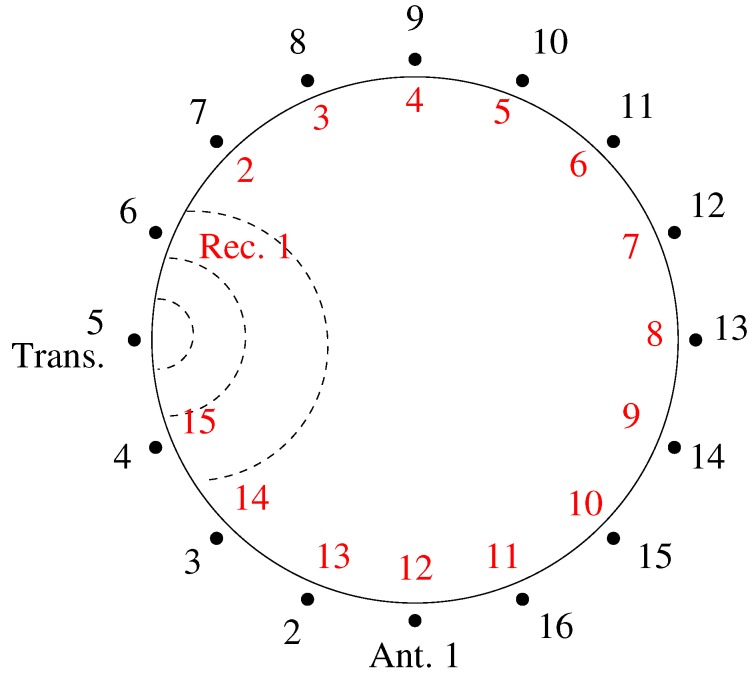
Schematic representation of the of the antenna numbering. The outer circle denotes the global node numbers (in black) used for the antennas when transmitting. The inner circle denotes the local node numbers (in red) used for the antennas when receiving, counting from the transmitter. In this example, Antenna 5 is transmitting.

**Figure 6:**
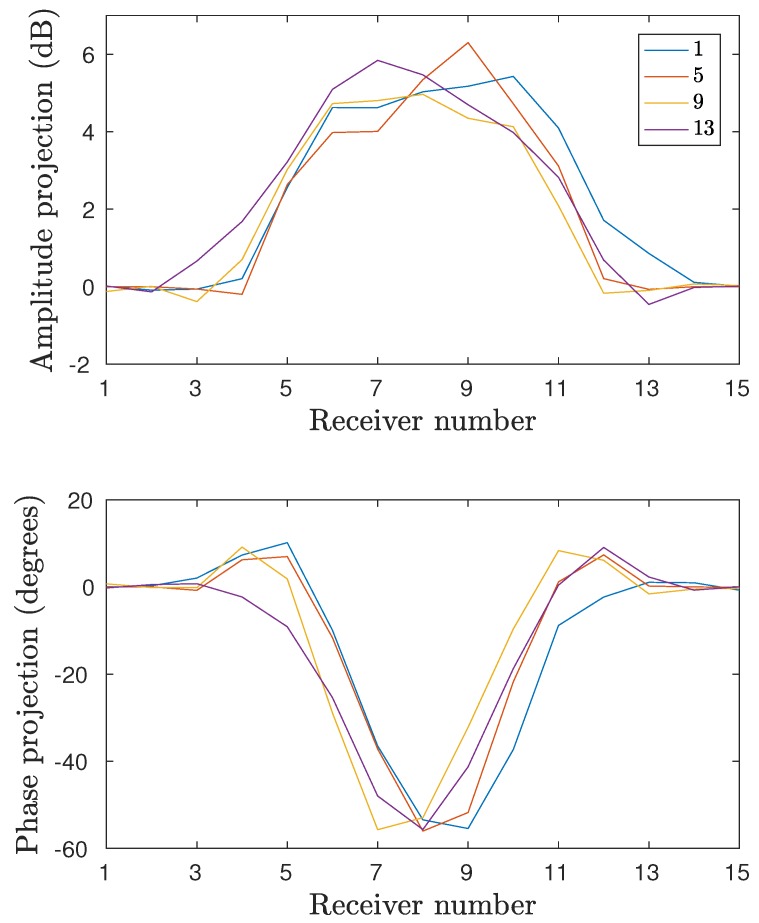
Projection data for four different transmitters: Antennas 1, 5, 9, and 13. Data are acquired at fourth layer and the inner shell is filled with the surrounding 80:20 glycerin–water mixture.

Figure 5 shows a schematic diagram of the 2D measurement configuration. The data are presented in projection form with respect to the local receiver numbers. For example, the 15 relative receiver numbers for Transmitter 1 consist sequentially of Antennas 2–16. For Transmitter 5, the 15 relative receivers consist of Antennas 6–16, followed in order by Antennas 1–4. Figure 6 shows the phase projections at 1500MHz and Layer 4 for Transmitters 1, 5, 9, and 13 for measurement series D where only the fibroglandular shell is present and the 80:20 glycerin:water mixture is used for liquid inside and outside of the plastic shell. In this case, the phase projections are essentially all in the negative direction which generally corresponds to a strongly lower permittivity object than that of the background. In fact, if the plastic were to have no impact, these measurement projections would be zero for all receivers. While the shape and location of the principle parts of each projection vary as a function of the object since it is not symmetric and not located exactly in the center of the target zone, the overall size and magnitude of the greatest portions of the projections are quite similar from all directions. This has been a consistent feature of this imaging configuration and has been exploited in previous studies [39]. Primarily, it indicates that a measurement from a single transmitter is sufficient to provide a representative example of the projections from all directions. Both amplitude and phase projections approach zero for the receive antennas closest to the transmitter (1–3 and 13–15).

**Figure 7:**
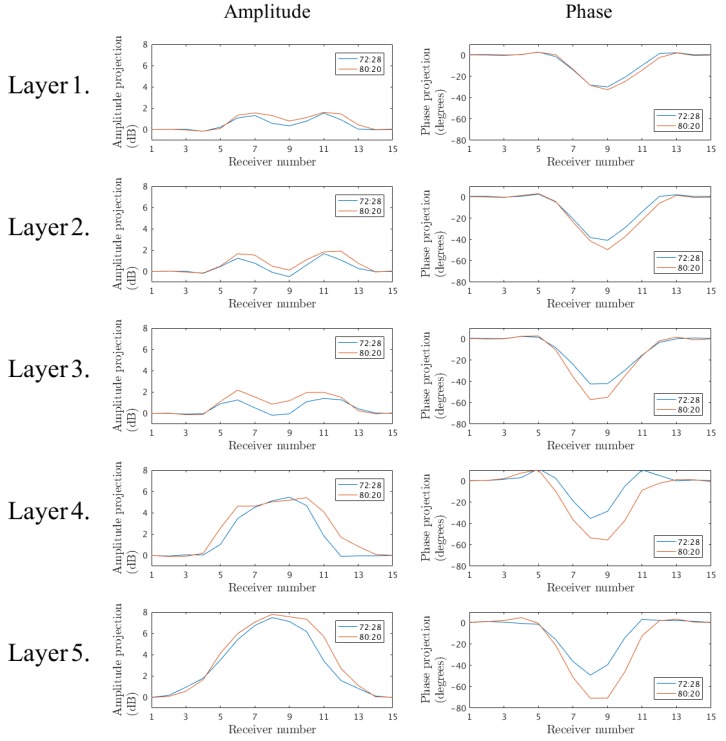
Projection data of the signals transmitted from Antenna 1 at 1500MHz for the five interrogated layers. Measurements of the inner shell filled with the 72:28 and 80:20 glycerin–water mixtures, respectively.

Figure 7 shows the amplitude and phase projections at 1500MHz for Antenna 1 for series C and D (the inner part filled with 72:28 and 80:20 glycerin–water mixtures, respectively) for the five interrogated layers. From these plots it is clear that there are significant similarities between the data from series C and D. For the lower layers, the projections are virtually identical, despite the different liquids. This is largely due to the fact that plastic constitutes a quite high proportion of the cross-sectional area. At higher layers, the measurements deviate more but the similarity is still substantial. It is also worth noting that, due to the 72:28 solution of series C having a higher permittivity than the 80:20 coupling bath, the phase would be expected to be positive. However, the plastic has a permittivity low enough to cancel this and in fact yields an overall negative phase shift. It is clear that the higher permittivity interior liquid appears to increase the phase for the 72:28 solution case but it is insufficient to overcome the effects of the plastic shell. Similar observations can be made for the amplitude projections.

**Figure 8:**
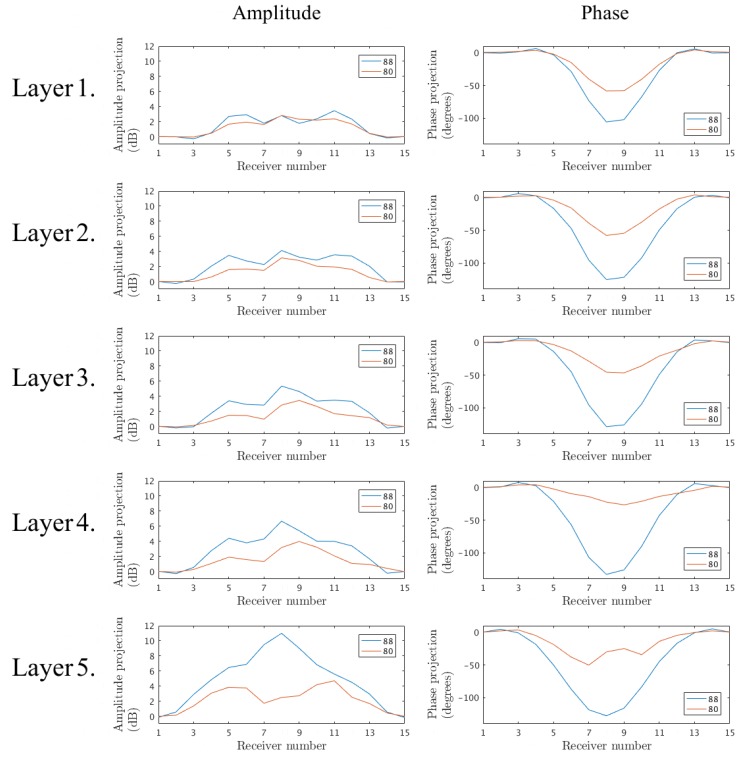
Projection data of the signals transmitted from Antenna 1 at 1500MHz for the five interrogated layers. Measurements of the outer shell filled with the 88:12 and 80:20 glycerin–water mixtures, respectively.

Similarly, Figure 8 shows the corresponding amplitude and phase projections for series A and B, where only the outer shell is present and the inner region is comprised of 88:12 and 80:20 glycerin:water mixtures, respectively. The trends are similar to those above, where the phase projections for just the plastic layer are quite significant in the negative direction. The phase projections for the 88:12 cases increase further in the negative direction, as would be expected, because the permittivity of the inner liquid is also less than that of the background. The proportional cross-sectional area occupied by the plastic is considerably less than that for the inner chamber. This percentage also decreases as the layers progress for Layer 1–5. In addition, the size of the object (in this case the area enclosed by the plastic shell) is considerably larger than that for the inner chamber. Consequently, it would be expected that the interior liquid would have a greater impact than that for previous case. However, the impact of the outer shell is still considerable both with respect to the phase and amplitude.

**Figure 9:**
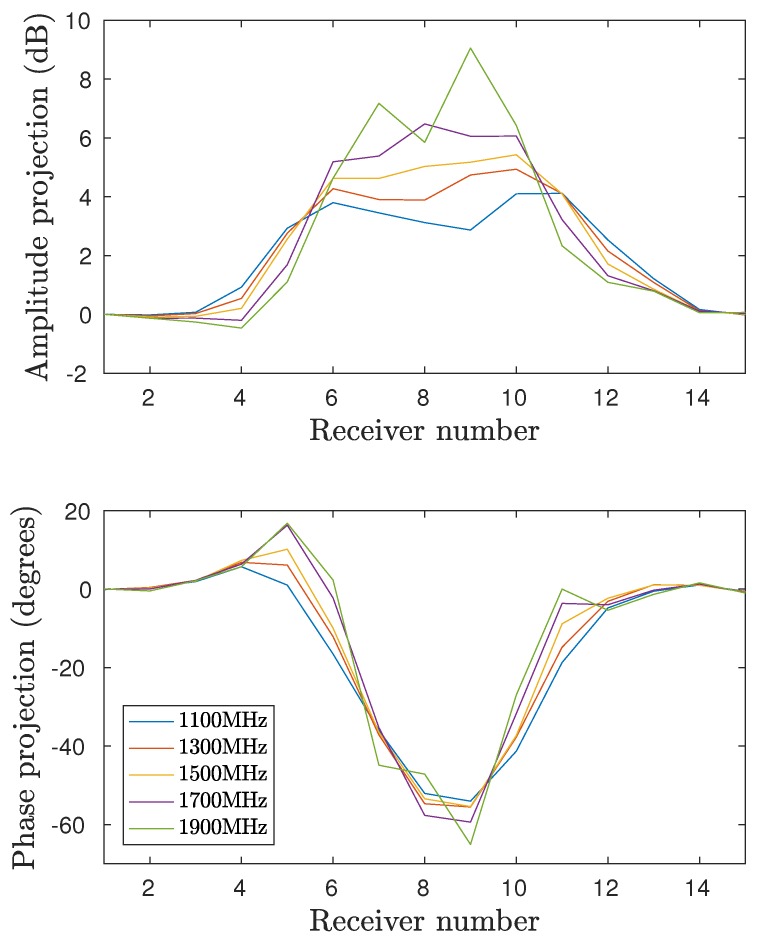
Projection data as function of receiver for the frequencies 1100MHz, 1300MHz, 1500MHz, 1700MHz, and 1900MHz. Antenna 1 is transmitting and the fourth layer is illuminated.

Finally, it is important to examine the measurement behavior with respect to the operating frequency. Figure 9 shows the amplitude and phase projections for series D, Layer 4 and Antenna 1 for a range of frequencies. It is worth noting that the phase projections are fairly constant with respect to frequency. In all cases, the impact of the plastic is consistently large across this considerable bandwidth.

### 3.2. Image Reconstructions

**Figure 10:**
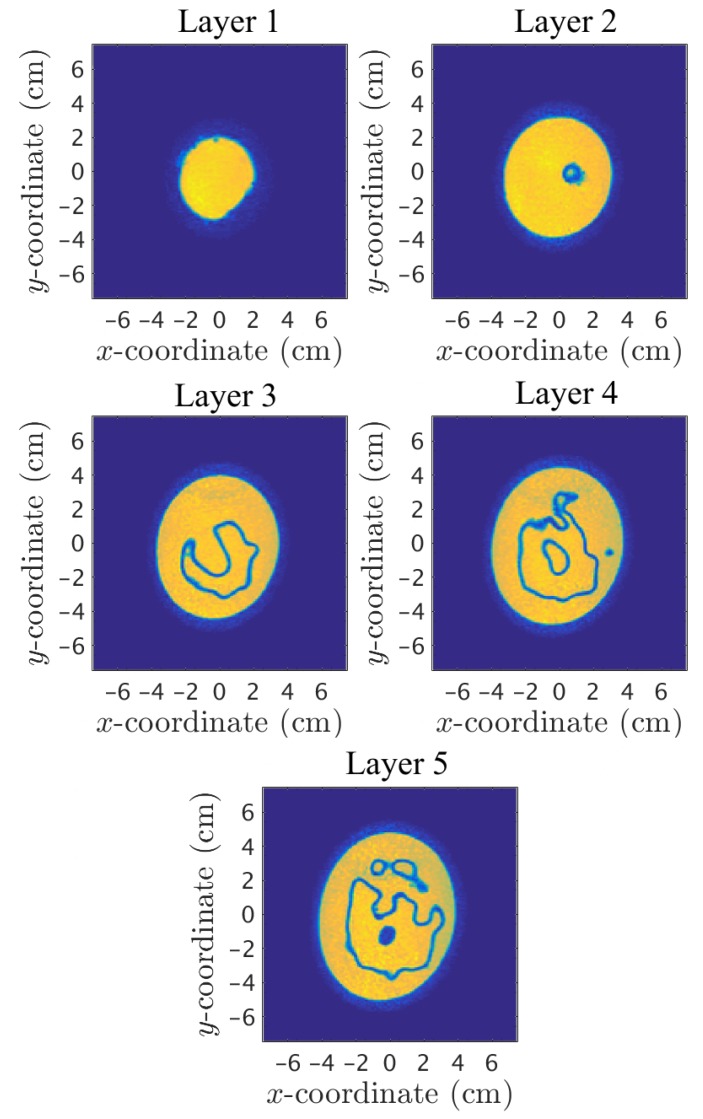
MRI scans of the five imaging layers.

By investigating the coronal planes of the 3D MRI, it was possible to study the amount of plastic in the inner chamber. This was determined by using a custom graphical tool which allows us to manually discretize a boundary of an image using a computer mouse, from which the software automatically computes the area within it. The ratio of plastic compared to the total coronal cross-sectional area of the inner chamber for the fibroglandular zone ranges from 14% at the lowest to 100% at the highest with an average of 26%. The plastic of the fibroglandular piece thus forms a significant part of the total cross sectional area of the phantom. MRI images for the associated layers are presented in Figure 10, where the two chambers were filled with water for visibility purposes in the MR images due to its high contrast with the low permittivity plastic.

**Figure 11:**
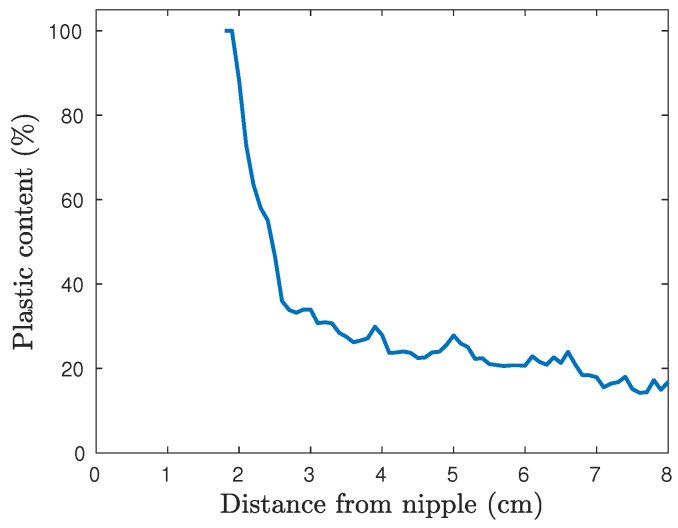
Plastic surface percentage of the cross sectional coronal planes of the fibroglandular shell as function of distance from the nipple.

As shown in Figure 10, the cross-sectional area, and thus the plastic percentage, varies significantly between the different layers. In Figure 11, the plastic percentage of the fibroglandular part is plotted as function of vertical position, i.e., distance from the nipple. This inner shell does not reach all the way to the nipple and thus the data here start at 1.8cm.

**Figure 12:**
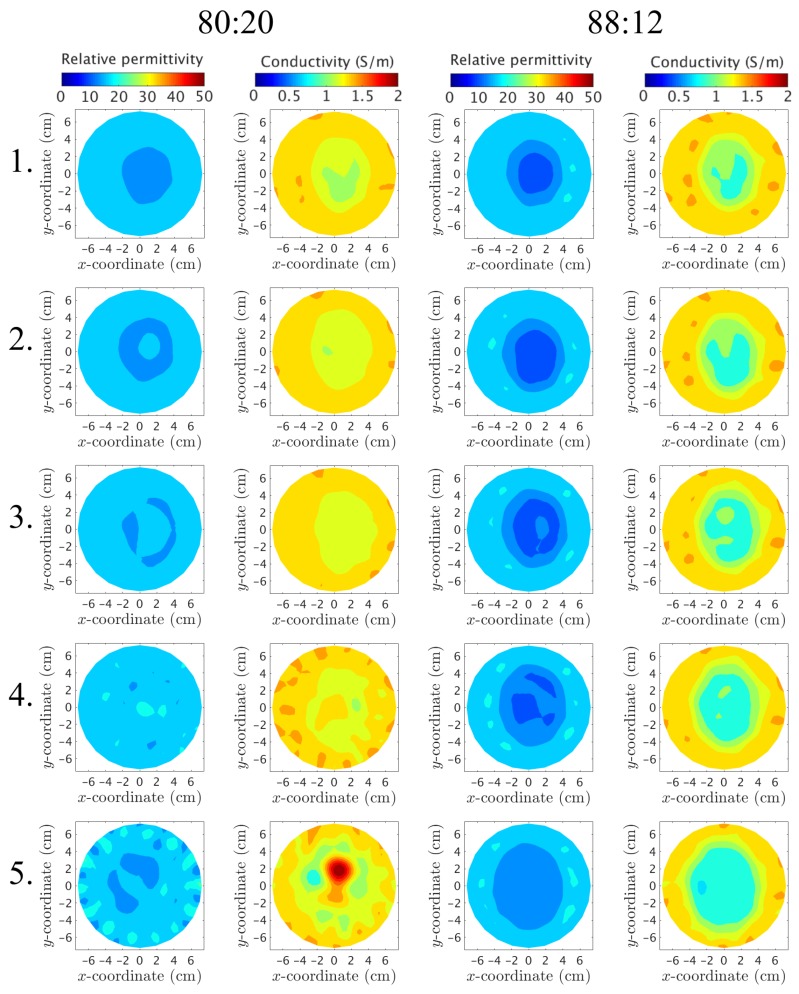
Reconstructed images at 1500MHz of the outer chamber. Each row depicts a layer of the phantom, starting from Layer 1 (closest to the nipple) up to Layer 5 (closest to the chest wall). The columns correspond to (from left to right) the permittivity using the 80:20 mixture, the conductivity for 80:20, the permittivity for 88:12, and the conductivity for 88:12, respectively.

Were the plastic to have no effect on the measurements, the recovered images would only depict a homogeneous bath when the phantom is filled with the surrounding liquid. Figure 12 shows the reconstructed permittivity and conductivity images at 1500MHz, where only the outer part is used and filled with the 88:12 (series A) and 80:20 mixtures (series B), respectively. Clearly, the plastic of this outer piece has only a minimal effect on the permittivity images, especially at Layers 3 and 4, whereas there is still some shadow remnants present along with artifacts around the edges in the conductivity images. For these particular layers, the plastic is only present in a small percentage of the imaging plane. This is consistent with the measurement data from the previous section. Conversely, for layers closer to the nipple, the images clearly show that something is present in the imaging plane. This is especially evident at Layer 1 and presumably occurs since a larger part of the imaging plane is now comprised by plastic due to the shape of the phantom. The large elevated property object in the conductivity image for Layer 5 along with the increased artifacts on the edges of both images for Layer 5 are most likely due to the antennas being positioned relatively close to the air–liquid interface where multi-path signal reflections are more prevalent than for other layers.

**Figure 13:**
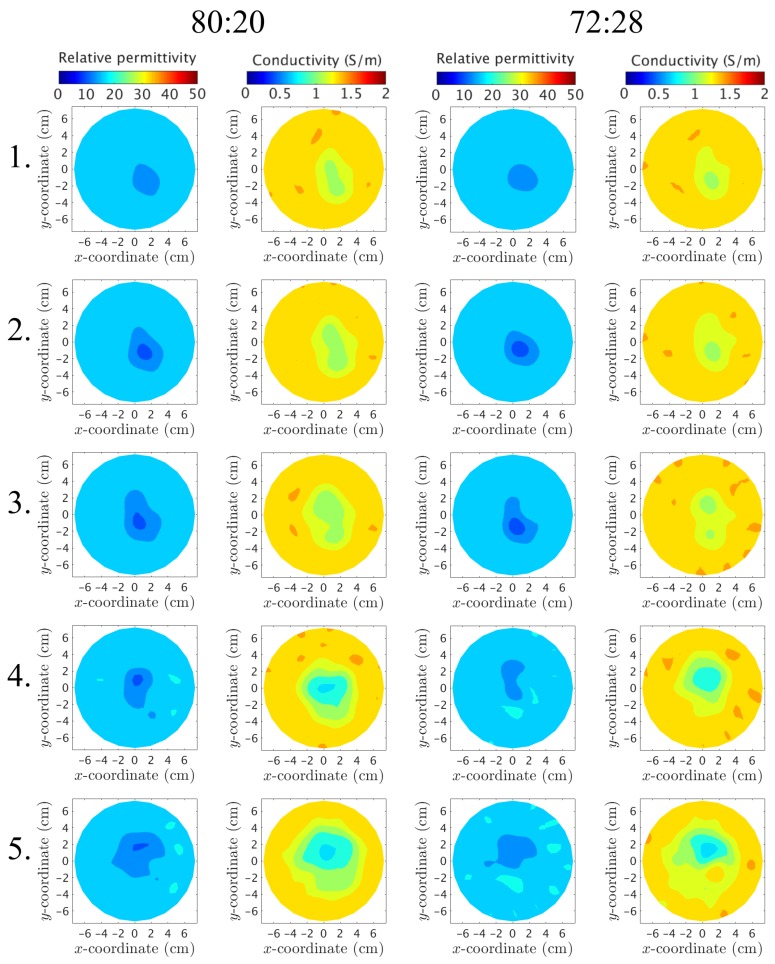
Reconstructed images at 1500MHz of the inner chamber. Each row depicts a layer of the phantom, starting from Layer 1 (closest to the nipple) up to Layer 5 (closest to the chest wall). The columns correspond to (from left to right) the permittivity using the 80:20 mixture, the conductivity for 80:20, the permittivity for 72:28, and the conductivity for 72:28, respectively.

Similarly, the inner plastic piece is reconstructed at 1500MHz and presented in Figure 13 for the 80:20 and 72:28 mixtures, respectively. The piece is visible at all layers for both the permittivity and conductivity cases. This piece has a “wrinkled” irregular shape that leads to a relatively high proportional plastic content at each layer.

For both phantoms and for all layers, the recovered object for the permittivity has properties less than that of the background. This is consistent with earlier observations that demonstrated strong correlation between negative phase projections and lower property value recovered objects [39]. In this situation, this negative property contribution can only be attributed to the low permittivity of the plastic. In addition, the permittivity object is virtually identical for the two different internal liquids for layers L–3. These correspond to plastic proportions of 51%, 35% and 26%, respectively (taken as averages over a 2 cm thick portion of the MR images surrounding each layer). It is clear that, while the plastic plays a somewhat minor proportion for the overall composition, its very high contrast with respect to the properties of those of the two liquids results in an outsized influence on the effective field measurements. The permittivity images for Layers 4 and 5 are still quite similar for the two liquids, but differ slightly in regards to the shape and size. These observations are consistent with the measurement analysis in the previous section. The conductivity images demonstrate similar trends to that of the corresponding permittivity images. The recovered objects all exhibit property values consistently less than that of the two liquids—the conductivity of the plastic is nearly 0.0 S/m. In this case, the plastic has the predominant influence for driving the recovered properties down. There is slightly more variation between the conductivity images for the two interior liquids than the permittivity cases, but the difference is fairly inconsequential.

## 4. Discussion

The GeePS-L2S phantom has proved the possibility of producing geometrically realistic phantoms via 3D printing. The intricate shape of the phantom captures the features of a real human breast, in both exterior and interior.

The main rationale for developing a 3D printed structure is to provide a stable and versatile universal phantom for which different groups can compare results. This particular incarnation does not degrade over time and its hollow structure allows for great variability by changing its content. Conversely, the printing material does not appear to possess the dielectric properties that are conducive to these types of experiments. Molded gel based phantoms do not exhibit this problem but, alternatively, are not stable over longer periods.

The GeePS-L2S phantom is a step towards a practical, realistic anthropomorphic breast phantom. However, this study has identified issues related to the large dielectric-property contrast ratios between the plastic and the phantom liquids. The dielectric properties of the plastic are quite low compared to those of the remaining imaging zone and thus has a sufficient impact on the scattered signals to such an extent that they can essentially form an image on their own. This further indicates that interior structures will be hard to image. This is especially apparent for the inner structure, for which the regular measurement was nearly indistinguishable from the control measurement when filling the plastic shell with the surrounding coupling medium. In order for 3D printed phantoms to be useful alternatives to other phantoms, the issue of the relatively thick, high contrast plastic needs to be addressed. Potential ways to accomplish this include either identifying a suitable material with properties closer to those of the tissue-mimicking liquids or developing new processes for generating thinner shells. An alternative option could be to use the 3D printed structure as a mold for gel based phantoms rather than as the actual phantom itself.

A 3D printable alternative with properties closer to those of biological tissue could be the conductive ABS plastic used by Faenger et al. [41]. One could also argue for a simplified phantom. Although an anthropomorphically realistic phantom is desired, signals at low microwave frequencies are generally not able to fully capture all of the fine details because of their long wavelengths. It should be possible to fabricate a more simplistic phantom that still resembles a human breast.

While the 3D-printing capability is compelling for being able to generate physically accurate representations of actual body structures, constraints such as the printing material need to be closely examined with respect to their influence and actual measurements need to be carefully evaluated. Development of the GeePS-L2S breast phantom was a necessary exercise to establish bounds on current technological capabilities. There is still considerable effort and innovation required before a fully functional phantom is available for realistic testing.

Finally, the phantom has only been tested with one particular system for these results. It was found that the plastic has a significant effect on the obtained reconstructions and it is necessary to understand this effect when interpreting their accuracy. It should further be emphasized that, although this study has pointed out certain issues regarding the high plastic content, this single study can neither reject nor confirm the general usefulness of the phantom. The authors would like to encourage other research groups to conduct similar studies. The phantom has also been tested with one particular choice of tissue mimicking properties. When studying the adipose region, the content can be varied to account for different radiographical densities. However, this study has shown that care must be taken when designing a phantom. Hopefully, the findings in this study will be useful for the community to develop and build even better phantoms in the future.

## Figures and Tables

**Table 1:** Glycerin content (volume percentage) of the two chambers for the different measurement series. An asterisk (*) denotes that this part was not included for the particular series.

Series	A	B	C	D
Outer chamber	88	80	*	*
Inner chamber	*	*	72	80
